# Greenhouse Gas Emissions from Molten Fluoride Electrolysis Composed of Raw and Magnet Recycling Derived Oxides: A Comparative Study

**DOI:** 10.3390/ma18010184

**Published:** 2025-01-04

**Authors:** Vesna S. Cvetković, Nataša M. Petrović, Laras Prasakti, Dominic Feldhaus, Srecko R. Stopic, Bernd Friedrich, Jovan N. Jovićević

**Affiliations:** 1 Institute of Chemistry, Technology and Metallurgy, National Institute of the Republic of Serbia, University of Belgrade, Njegoševa 12, 11000 Belgrade, Serbia; vukicevic@ihtm.bg.ac.rs (N.M.P.); jovicevic@ihtm.bg.ac.rs (J.N.J.); 2 IME Process Metallurgy and Metal Recycling, Institute of RWTH Aachen University, Intzestrasse 3, 52056 Aachen, Germany; lprasakti@metallurgie.rwth-aachen.de (L.P.); bfriedrich@metallurgie.rwth-aachen.de (B.F.); 3 TRIMET Aluminium SE, Aluminiumalle 1, 45356 Essen, Germany; dominic.feldhaus@trimet.de

**Keywords:** fluoride-based electrolyte, anode processes, RE electrolysis, magnet recycling derived oxides (MRDOs), in situ FTIR analysis

## Abstract

In situ measurements of the chemical identity and quantity of anode gases during electrochemical measurements and rare earth (RE) electrolysis from fluoride-based molten salts composed of different kinds of rare earth oxides (REOs) were performed using FTIR spectrometry. Linear sweep voltammetry (LSV) was carried out to characterize oxidation processes and determine the anodic effect from NdF_3_ + PrF_3_ + LiF + REO melt. RE complex formation and subsequent reactions on the GC anode surface were discussed to understand the formation pathways of CO/CO_2_ and perfluorocarbon gases (PFC), mainly CF_4_ and C_2_F_6_. The LSV shows that increasing the REO content from 1 wt.% up to 4 wt.% in the system, leads to a positive shift in the critical potential for a full anode effect, recorded around 4.50 V vs. W with 4 wt.% REO. The FTIR results from on-line off-gas analysis during LSV measurements indicate that the anode gas products were composed mainly of CO and CO_2_, whereas CF_4_ can be detected before the full anode effect and C_2_F_6_ at and after this phenomenon. Compositions of off-gases from electrolysis performed using different kinds of REOs were compared. The main off-gas component was found to be CO in RE electrolysis with REOs as raw materials, while in electrolysis with magnet recycling derived oxides (MRDOs), CO_2_ content was slightly higher compared to CO. PFC emissions during RE electrolysis were generally similar: CF_4_ was detected periodically, but in negligible concentrations, while C_2_F_6_ was not detected.

## 1. Introduction

Rare earth elements (REEs) have become key components in diverse crucial products in the green technology sector, central to the development of renewable energy and low-carbon technologies [1,2,3]. This trend is expected to continue, mainly due to the significant investment in clean energy initiatives [4]. Intensive and large-scale REE exploration is a waste-generating process that creates severe environmental issues [1,5], forcing industrialized countries to turn to alternative resources for rare earths, e.g., recycling these elements from REE-containing end-of-life (EOL) products [6,7,8]. As an essential direction for the development of clean, efficient, and simple extraction methods for the REEs from available resources, especially from end-of-life magnets, the molten salt electrolysis (MSE) approach is becoming the leading process. Electrochemical deposition technology uses electrode reactions to achieve separation and extraction of a single metal from complex electrolyte systems. The fundamental prerequisite for RE electrolysis and their alloys’ deposition is the applicability of chloride or fluoride-based molten salt electrolytes using RE oxides as raw materials [9]. Fluoride electrolytes provide better solubility of rare earth oxides than chloride ones, but both electrolyte media are involved in the consumption of the anode, producing greenhouse gases (GHGs) [10,11]. Despite numerous efforts, a comprehensive understanding of the environmental impacts associated with the production of REEs remains elusive [12]. Recovered RE elements from end-of-life magnets represent a small contribution to the overall RE supply chain today. However, as demand is rapidly increasing, the key will be to develop an economically viable recycling process [13]. Based on this background, our work in this field over the last decade led to the development of a new option for the recovery of REEs from NdFeB magnet scrap using a combination of pyrometallurgical treatment of spent NdFeB magnets and a subsequent molten salt electrolysis process. To complete complex REE recovery processes from EOL materials, fundamental knowledge of the mechanism of REE reduction processes and selective REE electrodeposition in an oxide–fluoride-based molten salt is of great importance [10,14,15,16,17,18]. Our approach of combining experiments and theory enabled us to construct a ternary phase diagram for the liquidus temperatures of the chosen fluoride-based molten salts, NdF_3_ + PrF_3_ + LiF [15]. Then, a dynamic model of the electrochemical process was developed to estimate the system variables and predict the anode effect using the Transfer Function (TF) estimation, Auto-Regressive with Extra inputs (ARX), Hammerstein–Weiner (HW), and Artificial Neural Network (ANN) identification methods [17]. In this way, the issues related to inappropriate composition and high melting temperatures of the mixture required for process realization were avoided. The next planned step was molten salt electrolysis using magnet recycling derived oxides (MRDOs) as a source of rare earth oxides [6]. With this particular approach, we succeeded in recovering REEs using MRDOs produced from used magnet scrap in the MSE using a fluoride-based molten salt electrolyte. However, we must emphasize the aspect of greenhouse gas emission (GHG) that could evolve on the glassy carbon anode during REE electrodeposition from fluoride melts. It is known that over time in RE electrolysis, the carbon anode is consumed, which uncontrollably increases the interelectrode gap and the cell voltage, periodically interrupting the process by replacing the anode, removing the cathode, etc. [19]. There are investigations about off-gas emissions in rare earth technology and significant improvements are expected to reduce PFC emissions [19,20,21,22]. To define the selective recovery of individual REEs from fluoride-based melts by electrochemical methods, we combined an electrolyte mixing REE + LiF + REO to optimize the deposition process and the purity of the final product [15]. The literature review shows no data reported on off-gases evolved during electrochemical measurements and RE electrolysis from fluoride-based melts composed of MRDO. This study aims to determine how the concentration of REO in the fluoride melts influences the critical potential of the full anodic effect and CO/CO_2_ and perfluorocarbon formation during RE electrolysis. The anode gas composition during electrochemical measurements and RE electrolysis from fluoride molten salts with different REOs will be analyzed to address more sustainable pathways for selective REE recovery.

## 2. Materials and Methods

### 2.1. Apparatus

**Figure 1:**
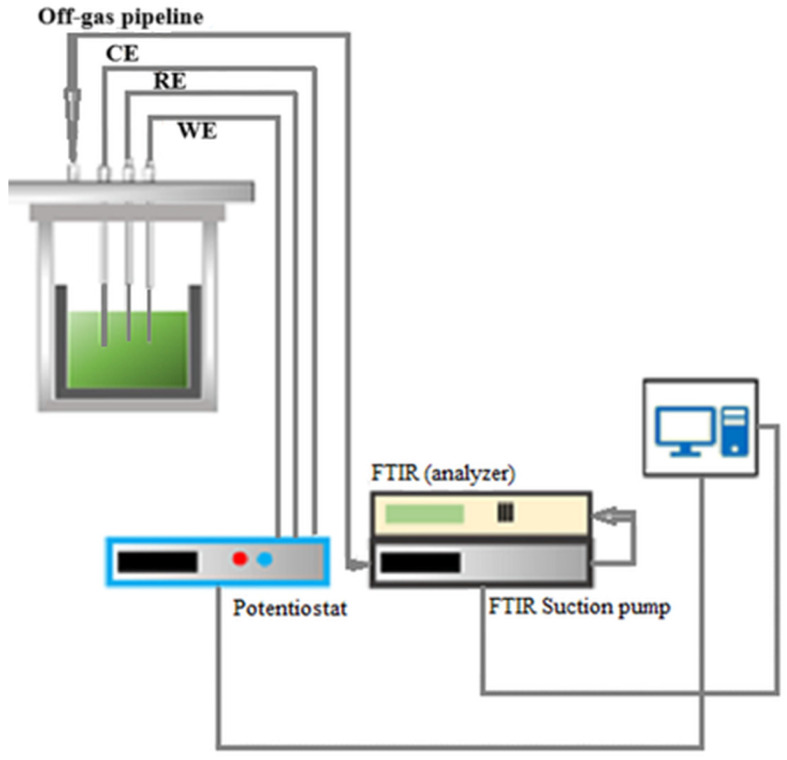
Schematic presentation of the experimental setup.

The electrochemical measurements were carried out in a graphite crucible, placed in a gas-tight stainless-steel cell with a water-cooled lid. The cell lid was cooled to protect the Swagelok system, which allowed the electrodes to be inserted during the experiments. In this way, the cell operates in an airtight atmosphere that prevents the electrolyte from oxidizing or harmful gases from leaking out of the cell. In addition to the holes for the electrodes, the lid has holes for the thermocouple (a thermocouple Type B), the argon flow (1.5 L/min), and the gas measurements. A crucible with the electrolyte was inserted into a stainless-steel container before the lid was put on and the experimental reactor was sealed (see Figure 1). Finally, the cell was placed in a resistance heating furnace and heated to 1323 K. The gases were extracted from the cell at a rate of 1.5 L/min using the exhaust gas analysis system. The off-gas analysis was performed every 5 s. During the electrochemical measurements and MSE, the off-gassed composition was in situ-monitored by FTIR.

### 2.2. Electrolyte Preparation

Lithium fluoride (LiF, 99.5% purity), neodymium fluoride (NdF_3_, 99.9% purity), neodymium oxide (Nd_2_O_3_, 99.9% purity), praseodymium fluoride (PrF_3_, 99.9% purity), and praseodymium oxide (Pr_6_O_11_, 99.9% purity) were purchased from Treibacher, Althofen, Austria. The fluoride-based electrolyte was homogenized according to the electrolyte preparation procedure, which has already been reported in detail [14]. Seven hundred and fifty grams of the fluoride-based melt mixture was placed in a graphite crucible and the experiment was set up according to the method described above. Powdered Nd_2_O_3_ and Pr_6_O_11_ as a source of the corresponding RE ions were added directly to the melt, while MRDOs were prepared from EOL magnets. The fluoride-based electrolyte composition, 61.2 wt.% NdF_3_ + 26.3 wt.% PrF_3_ + 12.5 wt.% LiF, was selected based on our previous tests [14], except that the main difference in the composition of the fluoride-based electrolytes lies in the origin of REO materials. The MRDO was produced from end-of-life NdFeB magnets through oxidation in air and subsequent carbothermic reduction under an 80 mbar Ar gas atmosphere [6]. With roughly 33 wt.% Nd and 10 wt.% Pr in the MRDO, it was a good basis for using this material in REEs as a source of REO [6]. In addition, the amount of MRDO used for the electrochemical measurements was also varied.

### 2.3. Electrode and Instrumentation

A three-electrode configuration was used in the electrochemical experiments, with the electrodes and thermocouple positioned in the molten fluoride in a custom-built stainless-steel cell. Once the electrolyte was melted, the electrodes were immersed in the melt and connected to an IviumStat potentiostat (5 A/10 V; Ivium Technologies, Eindhoven, The Netherlands), which was used to run the electrochemical measurements and MSE. For the electrochemical measurements, the working electrode (WE) was a glassy carbon rod with a diameter of 4 mm (GC, >99.99% HTW SIGRADUR^®^ G). Molybdenum (Mo) wire with a 2 mm diameter (EWG 99.95%) was used as a counter electrode (CE) and the quasi-reference electrode (RE) was tungsten wire (W, EWG 99.9%), 2 mm diameter. As for electrolysis, the working electrode was Mo, the counter electrode was glassy carbon, and W was used as the reference electrode. In all measurements, the electrodes (WE, CE, and RE) were 1 cm immersed in the electrolyte. All potentials in this paper are reported in reference to the tungsten quasi-reference electrode. The quasi-reference W electrode used in the electrolyte containing NdF_3_ + PrF_3_ + LiF and the corresponding REO or MRDO provided a reliable potential. Before each measurement, the electrodes were polished thoroughly using SiC paper and then cleaned [14]. Electrolysis experiments were carried out in constant potentiostatic mode for up to 5 h, during which constant off-gas monitoring was maintained. Figure 1 shows a schematic presentation of the experimental setup. A Fourier transformation infrared spectrometer (FTIR, Gasmet DX4000 Ansyco, Karlsruhe, Germany) continuously measured evolved anodic gases in situ. A Gasmet DX4000 FTIR analyzer was used, consisting of an FTIR unit, a gas pump, an oxygen pump, and a control unit. Argon was used at a flow rate of 1.5 L/min, regulated by a mass flow controller, and the FTIR spectra were recorded at a resolution of 8 cm^−1^ with 10 scans per measurement. Concentrations in ppm were determined using Gasmet’s Calcmet software (DX4040), which applies the Beer–Lambert law to match measured spectra with a precalibrated reference library. Before each experimental run, the baseline spectrum was analyzed using nitrogen to validate the optical path, ensure instrument stability, and correct for potential drift. The gas cell was maintained at 180 °C to prevent condensation, and periodic calibration checks ensured measurement accuracy. Both datasets, from the gas measurement (FTIR) and the electrochemical measurement (Iviumsoft 4.1178), were matched manually. The starting time of each experiment corresponding to the starting points of FTIR measurements were coordinated to create the voltammograms.

## 3. Results and Discussion

### 3.1. Off-Gas Emission During Electrolysis Using Raw RE Oxides

**Figure 2:**
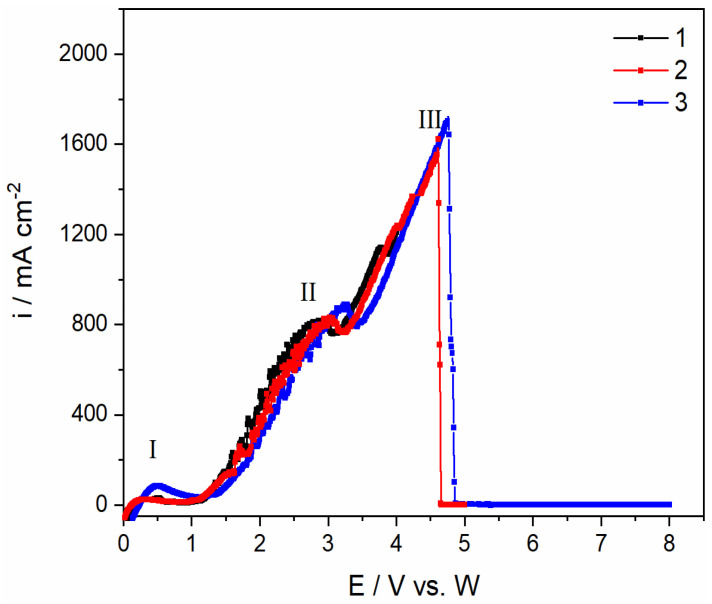
The linear sweep voltammograms recorded on a glassy carbon electrode in the NdF_3_ + PrF_3_ + LiF +2 wt.% Pr_6_O_11_ + 2 wt.%Nd_2_O_3_ electrolyte at final potentials: (1) 4.00 V; (2) 5.00 V; and (3) 8.00 V vs. W; T = 1323 K.

The electrochemical oxidation processes occurring on the GC electrode from the NdF_3_ + PrF_3_ + LiF + 2 wt.% Pr_6_O_11_ + 2 wt.%Nd_2_O_3_ electrolyte were investigated by linear sweep voltammetry. Typical LSVs recorded on a GC anode in the NdF_3_ + PrF_3_ + LiF +2 wt.% Pr_6_O_11_ + 2 wt.% Nd_2_O_3_ electrolyte were scanned in an anodic sweep from the equilibrium potential to different end potentials at a scan rate of 5 mV/s, as shown in Figure 2. According to Li et al. [23], the anode effect is mainly dependent on the applied potential and REO content in the melt. Aiming to deepen the study of anodic effect in the fluoride-based melts with Nd and Pr oxides present, three different end potentials were chosen. Within the scanned potential range, a relatively low scan rate was used to keep the system under pseudo steady-state conditions, similarly to a previous investigation [16]. During the anodic scan, a small oxidation current (I) at the potential ≈ 0.048 V vs. W could be assigned to the oxidation of the impurity and the maximum peak current density of ≈84 mAcm^−2^ recorded indicates a low level of impurities in the cell atmosphere [22]. In the potential range from 1.50 V vs. W up to 3.25 V vs. W, a sharp increase in anodic current is observed, ending with an anodic wave II at a potential of around 3.00 V vs. W and maximum current density of ≈ 820 mA cm^−2^. The increase in anodic current density should be attributed to the oxidation reactions of different oxyfluoride complexes formed and oxygen. For a molten salt electrolyte of a certain composition after the addition of Nd_2_O_3_ and Pr_6_O_11_, upon dissolution of the RE oxides, oxyfluoride complexes are formed. These complexes participate in subsequent reactions at the GC anode [10,24]. Based on the investigations carried out, it is very likely that the reactions in fluoride-based melts occur either by oxide or fluoride exchange with the fluoride or oxide complexes present in the electrolyte, Equations (1) and (2) [10,17,24,25]:

Nd_2_O_3_ + 2F^−^ → 2NdOF + O^2−^ (1)

Nd_2_O_3_ + [NdF_6_]^3−^ + 9F^−^ → 3[NdOF_5_]^4−^ (2)

Data on the dissolution of Pr_6_O_11_ in corresponding fluoride melts are scarce [26]. It is known that praseodymium oxide, Pr_6_O_11_, consists of a mixture of Pr_2_O_3_ · 4PrO_2_, which dissolves and forms different oxyfluorides such as PrOF [16,26]. The solubility of praseodymium oxide in neodymium fluoride-based melts or in praseodymium fluoride melts is similar to neodymium oxide dissolution and this is supposed to be due to the similarities in the atomic structure of Nd and Pr [25].

Based on this, we can propose the anodic reaction for Pr_6_O_11_ in which oxygen is generated:

3[PrOF_5_]^4−^ − 6e^−^ = 3/2O_2_(g) + 3Pr^3+^ + 15 F^−^ (3)

Very small oscillations in the current density seen on the voltammograms at potentials up to 3.00 V vs. W are probably due to the adsorption process of oxidation products formed by the electrochemical reactions, Equations (4)–(6), and partial passivation by oxygen-containing species at the GC anode, without turbulence by gas evolution [10,22].

C_(s)_ + O^2−^ → CO (g) + 2e^−^ (4)

C_(s)_ + 2O^2−^ → CO_2_ (g)+ 4e^−^ (5)

Additionally, PrO_2_ oxide present in Pr_6_O_11_ spontaneously reacts with carbon, as given in Equation (6) [26]:

PrO_2_ + 2C → Pr + 2CO (g) (6)

As further potential scans in the positive direction increase from 3.42 V to 5.00 V vs. W, the current density increasing almost linearly, with the maximum current density reaching values of around 1800 mA cm^−2^. Significant current densities are due to simultaneous oxidation reactions of Nd and Pr oxyfluoride complexes that occur, Equations (1)–(3), followed by oxidation, Equations (4)–(6), and gas evolution. During the scanning in the positive direction from fluoride-based electrolyte containing REO, LSV scan showed that the oxygen ions on a graphite electrode produce CO and CO_2_ according to Equations (3)–(6), followed by the adsorption of the oxidation products on the electrode surface, and finally CO and CO_2_ gas evolution.

As the experiment proceeded and the potential was further scanned in the positive direction, the current suddenly dropped and reached a value of almost 0 mA, which was maintained until 8.00 V, the terminal anodic potential. The potential of around 4.50 V vs. W (III), at which the current drops to almost zero value (Figure 2), is known as the critical potential for a full anodic effect. This potential is also associated with the phenomenon of the anodic effect in aluminium electrolysis [21,24,27].

**Figure 3:**
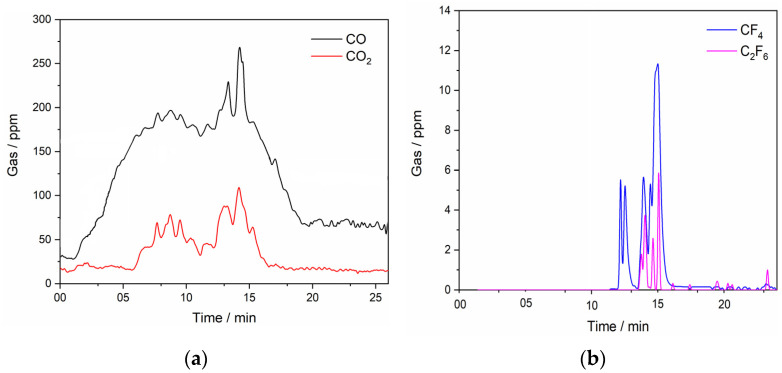
Off-gases recorded in situ with an FTIR spectrometer during LSV scan (anodic end potential 8.00 V, Figure 2) on GC electrode in NdF_3_ + PrF_3_ + LiF +2 wt.% Pr_6_O_11_ + 2 wt.% Nd_2_O_3_ electrolyte; T = 1323 K; (a) CO and CO_2_…(b) CF_4_ and C_2_F_6_ off-gases measured concentrations.

The anode gases measured with FTIR during the LSV experiment at the anodic end potential of 8.00 V vs. W were composed of CO, CO_2_, CF_4_, and C_2_F_6_, indicating that at potentials lower than the critical potential, CO appears to be the predominant off-gas component, Figure 3. As can be seen, the data derived from the FTIR results (Figure 3) are in correlation with the LSV in Figure 2. The results indicate that an amount below 50 ppm CO is always formed even if no potential is supplied to the system. This is related to the Boudouard reaction, which describes the formation of CO or CO_2_ as a function of the free energy of formation independent of the temperature. Below 1000 °C, mainly CO_2_ is formed, while above this temperature the equilibrium is switched to the formation of CO.

It is likely that at lower potentials, only oxygen-containing species are involved in the anode reactions and PFC emission starts at a higher potential, in this study most likely after 4.00 V vs. W [23]. CF_4_ emissions appear in off-gas products around the anodic effect, while C_2_F_6_ is detected at potentials slightly higher than the critical potential with a detection limit of about 6 ppm, indicating that fluoride species react with the graphite electrode and CF_4_ and C_2_F_6_ are evolved during the oxidation of fluoride ions, Equations (7) and (8) [22]:

4F^−^ (g)+ C (s) → CF_4_ (g) + 4e^−^ (7)

6F^−^ + 2C (s) → C_2_F_6_ (g) + 6e^−^ (8)

It is supposed that during the electrolysis more chemical or electrochemical reactions could take place on the anode and numerous unstable intermediate compounds such as COF_2_ or COF are formed and react further with GC, Equations (9)–(11) [27,28]:

2COF_2_ + C (s) → CF_4_ (g) + 2CO (g) (9)

4COF + C (s) → CF_4_ (g) + 4CO (g) (10)

Also, COF_2_ could spontaneously self-decompose (∆ G = −3 Jmol^−1^ at 1233 K) according to [27]:

2COF_2_ → CF_4_(g) + CO_2_ (g) (11)

In addition, when the praseodymium/neodymium oxide concentration near the anode becomes very low, the following two reactions proceed on the GC anode [26,28]:

PrF_3_ + 3/4C (s)→ Pr + 3/4CF_4_ (g) (12)

4NdF_3_ + 3C (g) → 4Nd + 3CF_4_ (g) (13)

To evaluate the gas emissions during the process, LiF used in this investigation should also be taken into account, as it acts as a dilution agent for the melts and a donor of F^−^ ions, and as such is involved in PFC formation [28]:

4LiF + C→4Li + CF_4_ (14)

6LiF + 2C (s)→6Li + C_2_F_6_ (g) (15)

It can be assumed that a combination of three gases, CO, CO_2_, and CF_4_, and probably additional gases like C_2_F_6_, COF, or COF_2_ build up a current-impervious gas film on the anode surface, leading to the passivation of the electrode and to a full anodic effect [29].

Previous comparable experiments have analyzed Nd or Pr oxide containing 0 wt.% [16] up to 4 wt.% in the electrolyte [14,16] under the same conditions. When compared to the results shown in Figure 2 and Figure 3, with similar results obtained in previous studies [16,21], the anode effect is shifted to a more positive potential, whose value is around 4.50 V vs. W at 4 wt.% REO. The higher REO concentration in the melt leads to more oxyfluoride complexes being formed, which are expected to prevent partial passivation of the GC electrode by oxygen-containing species at a lower anodic potential. This would imply that the rare earth oxide concentration would affect the electrochemical anode reactions, which would explain the shifts of the critical potential values for a full anodic effect towards more positive values [16,23].

**Figure 4:**
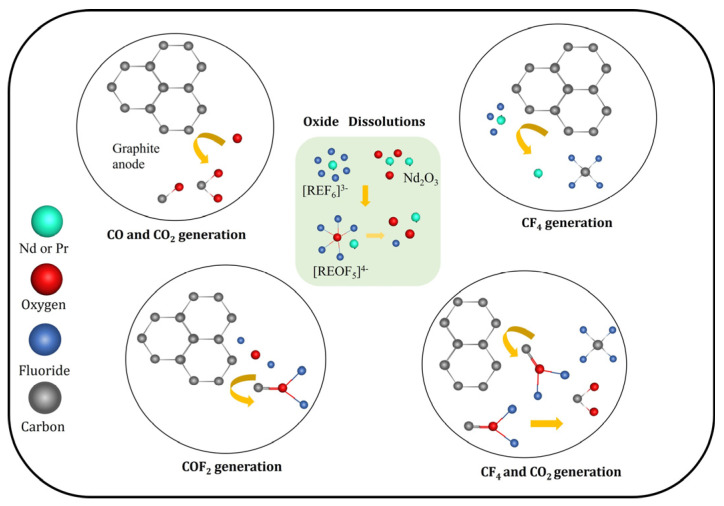
Schematic presentation of the proposed reactions on GC anode, including COF_2_ formation.

The reactions and their correlations on the GC anode surface and its immediate vicinity considered in this study during MSE from fluoride-based melts are summarized in Figure 4. As can be seen, we have assumed REO dissolution and two types of complexes formed, fluorides such as [REF_x_]^y−^ and oxyfluoride [REOF_x_]^y−^, which are involved in CO_2_/CO and CF_4_/CF_6_ formation. The other was an intermediate COF_2_, which is reactive with many common oxides, with a tendency to subsequently self-decompose [27].

**Figure 5:**
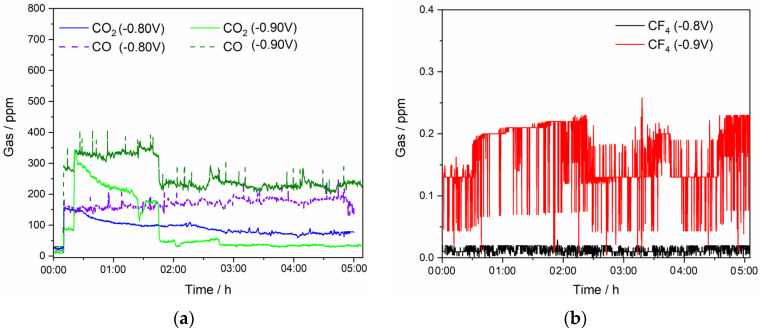
Off-gases generated on the GC anode recorded in situ with an FTIR spectrometer during potentiostatic deposition at different potentials: −0.80 V and −0.90 V vs. W applied: (**a**) measurements for CO/CO_2_ and (**b**) CF_4_ off-gas; working electrode Mo, in NdF_3_ + PrF_3_ + LiF +2 wt.% Pr_6_O_11_ + 2 wt.% Nd_2_O_3_ electrolyte; T = 1323 K.

To better understand the anodic reactions in the RE electrolysis processes, the deposition potentials and the electrolyte composition were adjusted to observe which anodic gases evolved. The composition of the anode gases evolved during RE electrolysis in NdF_3_ + PrF_3_ + LiF molten salt containing 2 wt.% of Nd_2_O_3_ and 2 wt.% of Pr_6_O_11_ at different potentials was analyzed, see Figure 5. The deposition potentials were selected based on our previous investigation. The off-gas measurements began at the time when the potential was imposed on the system, Figure 5a,b.

The anode gas products are mainly composed of CO and CO_2_, which is in accordance with previous reports [19,21,22,24,30]. There is an obvious difference in the CO and CO_2_ gas concentrations emitted during the electrolysis. The average CO concentration was approximately 200 ppm, while CO_2_ concentration was approximately 100 ppm for a deposition potential of −0.80 V vs. W, whereas for a deposition potential of −0.90 V vs. W, CO content was on average 250 ppm and CO_2_ concentration well below 100 ppm. After a certain deposition time, the quantity of CO_2_ became substantially smaller, which could be attributed to the partial passivation of the anode active sites with oxygen-containing ions. The continuous gas analysis shows that CF_4_ was detected in negligible concentrations. CF_4_ was detected periodically, and, when recorded, the highest concentration was around 0.2 ppm, while C_2_F_6_ is not detected. C_2_F_6_ is always formed after CF_4_ but in rather small amounts, just above or below the detection limit. Therefore, these data are not reliable and are not presented in Figure 5 or in the discussion. The potential of CO formation is 1.297 V, which is lower when compared to the one of CO_2_ (1.454 V), which is why it is probably formed first, resulting in a higher value in off-gas composition [31]. In addition, CO_2_ cannot react with the GC itself, but gas might penetrate into cracks and pores in the anode and the Boudouard reaction takes place there, Equation (16) [32]. Consequently, the concentration of CO in anodic gases is in agreement with the obtained results.

CO_2_ (g) + C(s) ⇌ 2CO (s) (16)

The RE electrolysis in this study was carried out at a controlled voltage significantly lower than the voltage imposing a full anodic effect, as seen in Figure 2. The formation of PFC gases represented by Equations (12)–(15) is not to be expected in this potential range. Therefore, the compatible anode voltage was controlled and the oxidation of fluoride ions to PFCs was not achieved. PFCs are formed at significantly higher anodic potentials than the potential associated with CO/CO_2_ formation, indicating that this approach minimizes the emissions of CF_4_ and C_2_F_6_ at the anode and reduces the global warming potential, Figure 5.

In the global context, this CO/CO_2_ and PFC emission rate is small compared to Al metal production [11].

Other parameters that could be important for the gas emissions in RE electrolysis, such as the applied current density at the anode, the composition of the electrolyte, the oxide concentration, the working temperature, etc., are elaborated in [17,24,33]. Aside from the development of non-consumable or dimensionally stable anodes (DSAs) [11], the molten salt electrolyte composition will be one of the ways to limit CO/CO_2_ and PFC emissions in the future. However, more sustainable ways should be found to keep the REE production process within strict environmental regulations.

### 3.2. Off-Gas Emission During Electrolysis Using Magnet Recycling Derived Oxides (MRDOs)

**Figure 6:**
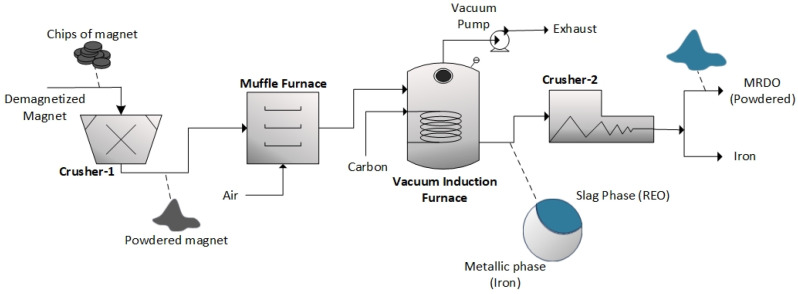
Schematic flow diagram of magnet recycling-derived oxide (MRDO) production from end-of-life NdFeB magnet.

In the future, the development of sustainable and economically viable recycling processes that enable the recovery of REEs reinserted into the supply chain will become an important source of the overall RE supply. We have proposed a combination of a pyrometallurgical process [34] and subsequent MSE from fluoride-based molten salts [6]. Figure 6 summarizes rare earth oxide production, referred to as magnet recycling derived oxides (MRDOs), produced directly from spent NdFeB magnets. The flow of the process is briefly depicted in Figure 6. The methods developed by [34,35] were adopted in this research to produce high concentrations of rare earth oxides. The process started with grinding the magnet into powder from its initial shape (small chips). The powdered magnet was then fed to a muffle furnace, where oxidation occurred. A vacuum induction furnace under an inert vacuum atmosphere was then utilized to process the oxidized magnet. Stoichiometrically calculated carbon powder was added to the magnet to perform carbothermic reduction. The addition of carbon was also to avoid the consumption of the graphite crucible used to contain the oxidized magnet during the process. Separation between the metallic phase (iron-rich) and slag phase (rare earth oxide-rich) was observed during the experiment, as shown in Figure 6. Mechanical separation was then carried out with a crusher at the end of the process flow to obtain the rare earth slag phase (MRDO) in the form of powder, which was easily separated from the metallic iron chunks. According to ICP-OES results, MRDO contains roughly 33 wt.% Nd and 10 wt.% Pr in different NdFeB-based rare earth oxides such as Dy_2_O_3_, NdO_2_, Nd_2_O_2_, Pr_5_O_9_, Nd_2_O_3_, Pr_2_O_3_, DyFeO_3_, and PrNdO_2_ [6].

The most commonly used electrolytes for the industrial production of RE metals and alloys in MSE are fluoride-based salts that are usually composed of rare earth fluoride, LiF, and REO dissolved in molten salts as a raw material for RE metal production.

**Figure 7:**
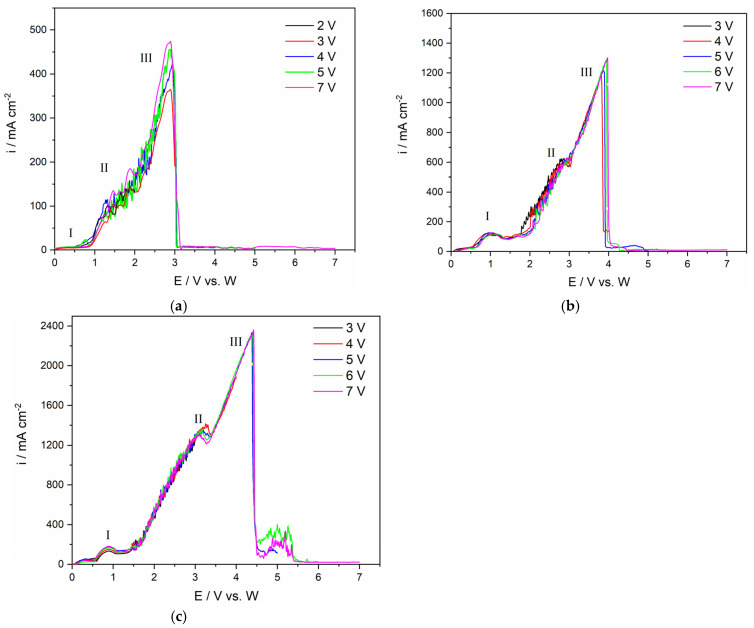
The linear sweep voltammograms recorded on a GC electrode in the NdF_3_ + PrF_3_ + LiF electrolyte containing (**a**) 1 wt.% of MRDO; (**b**) 3 wt.% of MRDO; and (**c**) 4 wt.% MRDO at different end potentials, T = 1323 K.

Following our previous results [14], the fluoride-based melt containing 1 wt.%, 3 wt.% or 4 wt.% of MRDO was selected for electrochemical measurements and RE electrolysis in this study. Figure 7 presents the LSVs recorded for different MRDO contents in the fluoride electrolyte.

For a direct comparison of electrochemical measurements, the basic electrolyte composition of 64.41 wt.% NdF_3_ + 21.37 wt.% PrF_3_ + 12.5 wt.% LiF was maintained. The voltammograms were scanned at 5 mV/s, consistent with previous experimental conditions. Magnet recycling derived oxide (MRDO) concentration in the electrolyte was the only controlled variable. The conditions for the experiment were selected to attempt to quantify the environmental impact of this process, since there are not many results regarding PFC emissions from the recovery of REEs from recycled magnets. As can be seen in Figure 7b,c, in the voltammograms presented, the oxidation current (I) starting at a potential around 1.00 V probably originates from the impurities in the electrolyte. In this particular case, the MRDO consists of different REOs accompanied by various iron/boron compounds. The oxidation of the oxygen ions generated by these impurities’ dissolution participate in the subsequent reaction in contact with the glassy carbon anode. The appearance of the anodic peak (II) in the voltammogram in Figure 7a recorded with 1 wt.% of MRDO added to the NdF_3_ + PrF_3_ + LiF electrolyte occurs at the potentials between 1.50 V and 2.00 V vs. W, with the oxidation peak current density ≈ 200 mA cm^−2^. An increase in oxidation current starting at about 2.00 V is also attributed to the oxidation of oxygen ions under gas evolution conditions until ≈ 3.00 V, Figure 7a. At the peak potential (III), the oxidation current density reaches 500 mA cm^−2^, before dropping to 0. This sudden drop in the current manifests the full anodic effect.

At 3 wt.% MRDO added to the electrolyte, Figure 7b, in the voltammograms, the peak current density (II) at potential ≈ 3.00 V vs. W reaches a value of around 650 mA cm^−2^. The current density then drops to a lower level, indicating a partial anodic effect, until the value rises again. Finally, the peak current density (III) increases and reaches 1300 mA cm^−2^, at an anodic peak potential around 3.50 V. The observed maximum current density at a potential around 3.50 V vs. W is followed by a sudden current fall, manifesting the full anodic effect; this time shifts slightly positively compared to the value in the LSV in Figure 7a. With 4 wt.% of MRDO in the fluoride-based melt, the maximum oxidation current density (III) at an anodic peak potential around 4.50 V, before dropping to 0, was about 2000 mA cm^−2^, which is a characteristic of the full anodic effect, as shown in Figure 7c.

The fact that the oxidation current density Increases with Increasing MRDO content in the fluoride-based electrolyte, and that the critical potential shifts towards more positive potentials, proves that the full anodic effect depends on the REO content.

**Figure 8:**
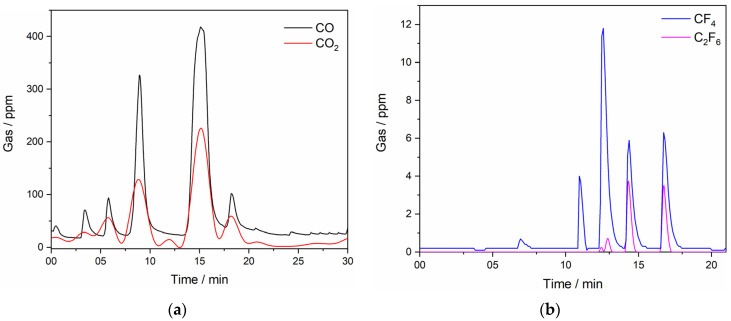
Off-gases generated on the GC anode recorded in situ with an FTIR spectrometer during LSV scans of Figure 7c anodic end potential 7.00 V vs. W, in NdF_3_ + PrF_3_ + LiF + 4 wt.% MRDO electrolyte; T = 1323 K; (**a**) CO and CO_2_…(**b**) CF_4_ and C_2_F_6_ off-gases concentrations measured.

The FTIR analysis of the anode gas composition during LSV measurements on a graphite anode in NdF_3_ + PrF_3_ + LiF molten salt containing 4 wt.% of MRDO is shown in Figure 8, where one exemplar measurement for clarity is presented. Once again, excellent agreement with our previous experiments is noted (Figure 3). FTIR results showed that until an anode effect occurs, CO and CO_2_ were the main off-gas components, with CF_4_ also detected in the anodic gases around the critical potential. The appearance of C_2_F_6_ in the anodic gases follows after CF_4_ evolution, showing the same tendency as in the RE electrolysis with raw REO, but at a significantly lower value of ≈ 3.5 ppm as the highest recorded value. To sum up LSV experiments, the anodic peak maximum current density at the glassy carbon anode, within the scanned potential range, especially between the partial and full anodic phenomenon, depends on the rare earth oxide concentration in the electrolyte. The same tendency can also be seen in the correlation between the peak potential and the fraction of CO/CO_2_ or PFC in the off-gas composition. This means that the RE oxides in MRDO are dissolved and the oxyfluoride complexes formed shift the critical potential values towards more positive potentials and disable the recognition of partial passivation by oxygen-containing species. This is similar to what was previously reported about the influence of REO on the anodic effect [24]. This is a further indication of the validity of MRDO used in RE electrolysis. When comparing LSVs, (Figure 2 and Figure 7c) with 4 wt.% of REO and MRDO added to the melt, the full anodic effect is around 4.50 V in both cases, indicating that MRDO can be used as an REE raw material in electrolysis. Based on these results, most of the REO from MRDO produced from spent magnets appears fully dissolved in the molten fluoride mixture. Dissolved species participate in the anodic processes, producing CO_2_/CO and CF_4_ in almost the same way as in RE electrolysis with raw REO. The only difference observed in the system with 4 wt.% MRDO is that the resulting current density increases again after the anodic effect, Figure 7c. Most likely, a very small area of the surface becomes gas-free for a short time, which is associated with PFC formation, leading to new gas bubbles again adsorbed on the anode, forming an insulating film.

**Figure 9:**
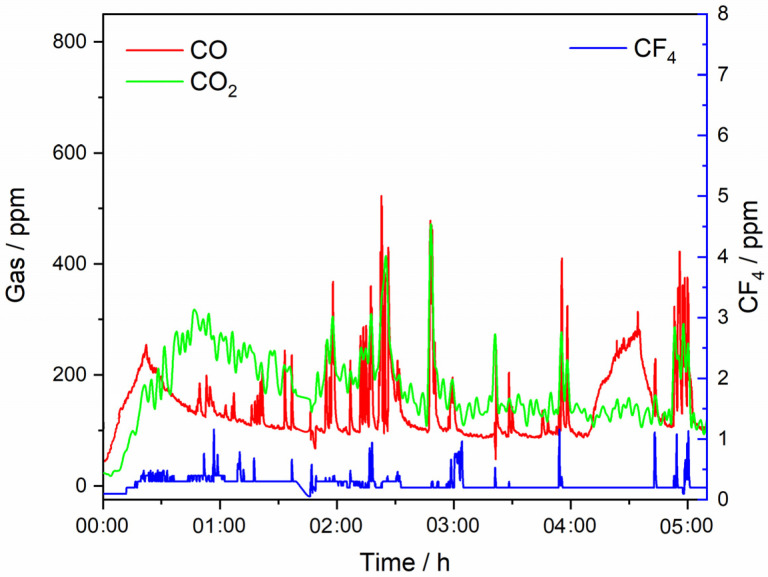
Off-gases generated on the GC anode recorded in situ with an FTIR spectrometer during potentiostatic deposition at potential of −0.90 V vs. W; working electrode Mo, in 64.41 wt.% NdF_3_ + 21.37 wt.% PrF_3_ + 12.5 wt.% LiF+ 4 wt.% MRDO electrolyte; T = 1323 K.

To quantify potential environmental impacts concerning greenhouse gas emission from the fluoride-based MSE process, we investigated the off-gas composition during REE extraction from NdF_3_ + PrF_3_ + LiF + 4 wt.% MRDO, Figure 9. Our previous experiments have shown that REEs can be successfully extracted by continuous electrolysis under certain process conditions [6]. This is only one average value of a minimum of three experiments involving laboratory measurements; therefore, the off-gas concentration values should be considered more for the qualitative description of the systems and processes than for quantification. However, for a sustainable process for RE electrolysis and RE metal production, it is still important to estimate emission factors that should at least minimize CO_2_ and PFC emissions.

The composition of the off-gases during RE electrolysis from fluoride-based melts with MRDO is qualitatively consistent with that observed in conventional RE electrolysis with REOs as raw materials. In the RE electrolysis experiments with MRDO added to the fluoride-based melt, only minor amounts (<20 ppm) of CO are present at the beginning of the process, Figure 8a, due to the consumption of the high-purity graphite anode due to the reaction, Equation (16). The results show that off-gases are composed of CO, CO_2_, and CF_4_, as seen in Figure 9. At the beginning of the electrolysis, the portion of CO_2_ gases in off-gases is a little bit higher than CO, showing the same tendency during the electrolysis, but with a significantly higher amount (approximately 200 ppm) than RE electrolysis with REOs as raw materials, where on average the CO_2_ concentration in off-gases is around 50 ppm. Conversely, the average value of CO in off-gases is approximately 100 ppm, significantly lower than in RE electrolysis (250 ppm) with REOs as raw materials. The content of CO/CO_2_ in off-gases remained relatively unchanged, showing the stability of the process. However, there were some spikes detected periodically, which depicted a significantly higher CO/CO_2_ concentration in off-gases, but never above 500 ppm. CF_4_ was detected in negligible quantities, as was the case in the previous electrolysis experiments. This further indicates that the electrodeposition of REEs within the applied potential range occurs at the expense of their corresponding oxides, provided by MRDO.

Some of the possible reasons may be due to the following: (i) CO_2_ is also formed as a consequence of a back reaction from dissolved oxides/metals from MRDO. (ii) Primary anode reactions and the Boudouard reaction proceed with chemisorbed C-O complexes as an intermediate step. The complexes formed can occupy almost the entire electrode surface without leaving free sites for the Boudouard reaction, leading to CO_2_ formation as the preferred reaction [36]. (iii) Various REOs and metals (M_i_) present as impurities in MRDO are dissolved and participate in forming various RE/M_i_-O-F complexes, which subsequently take part in simultaneous electrochemical anode reactions, working in favor of CO_2_. In addition, CF_4_ is also present in off-gases and some of the current is probably used for fluoride ion discharge, leading to a reduction in the rate of CO formation [21]. CO and CO_2_ are the main off-gas components, indicating that during the electrolysis at the chosen potential only oxygen-containing species are involved in anode reactions, Equations (4)–(6). However, this simplified picture is most likely not compatible with the complex multivalent redox transitions prevalent in REE extraction by MSE using MRDO. The agreement between the FTIR data of off-gas compositions within a range of REEs through raw REO and MRDO leads us to believe that the RE electrolysis process governed by MRDO obtained by the pyrometallurgical method captures the key electrolysis process that governs the REE extraction of end-of-life products.

## 4. Conclusions

The present study leads to the following conclusions related to greenhouse gas emission during REE extraction from fluoride-based molten salts composed of raw REO and REO from MRDO:

(i) It was demonstrated that the critical potential for a full anode effect is around 4.50 V at 4 wt.% raw or MRDO REO. Increasing the REO content from 1 wt.% up to 4 wt.% in the system leads to a more positive shift in the critical potential for a full anode effect.
(ii) The FTIR results from on-line off-gas analysis during LSV measurements displayed that the anode gas products were composed mainly of CO and CO_2_, whereas CF_4_ was detected before the full anode effect and C_2_F_6_ was emitted during and after this phenomenon.
(iii) The dissolution of REO in fluoride-based melts leads to the fluorides such as [REFx]^y−^ and oxyfluoride [REOFx]^y−^ complexes involved in CO_2_/CO and CF_4_/C_2_F_6_ formation on the anode. A schematic presentation that incorporates the complex formation and its subsequent reactions on the GC anode surface was developed.
(iv) The main off-gas component in RE electrolysis with REO as the raw material is CO. In contrast, the CO_2_ content was slightly higher than the CO content in electrolysis with oxides derived from magnetic recycling (MRDOs). PFC emissions during RE electrolysis were generally similar: CF_4_ was detected periodically, but in negligible concentrations, while C_2_F_6_ was not detected. It is likely that various REOs dissolved from MRDO are involved in back reactions occupying almost the entire electrode active surface without leaving free sites for the Boudouard reaction, leading to CO_2_ formation as the preferred reaction.
(v) The experimental results using LSV and FTIR measurements demonstrate that the future development of REE recycling from molten salts composed of MRDO is expected to be an innovative method due to its environmental impact benefits.

## Data Availability

The original contributions presented in this study are included in the article. Further inquiries can be directed to the corresponding authors.
